# Machine learning-based prediction of critical illness in children visiting the emergency department

**DOI:** 10.1371/journal.pone.0264184

**Published:** 2022-02-17

**Authors:** Soyun Hwang, Bongjin Lee

**Affiliations:** 1 Department of Pediatrics, Severance Children’s Hospital, Yonsei University College of Medicine, Seoul, Korea; 2 Department of Pediatrics, Seoul National University Hospital, Seoul, Korea; University of Maribor, SLOVENIA

## Abstract

**Objectives:**

Triage is an essential emergency department (ED) process designed to provide timely management depending on acuity and severity; however, the process may be inconsistent with clinical and hospitalization outcomes. Therefore, studies have attempted to augment this process with machine learning models, showing advantages in predicting critical conditions and hospitalization outcomes. The aim of this study was to utilize nationwide registry data to develop a machine learning-based classification model to predict the clinical course of pediatric ED visits.

**Methods:**

This cross-sectional observational study used data from the National Emergency Department Information System on emergency visits of children under 15 years of age from January 1, 2016, to December 31, 2017. The primary and secondary outcomes were to identify critically ill children and predict hospitalization from triage data, respectively. We developed and tested a random forest model with the under sampled dataset and validated the model using the entire dataset. We compared the model’s performance with that of the conventional triage system.

**Results:**

A total of 2,621,710 children were eligible for the analysis and included 12,951 (0.5%) critical outcomes and 303,808 (11.6%) hospitalizations. After validation, the area under the receiver operating characteristic curve was 0.991 (95% confidence interval [CI] 0.991–0.992) for critical outcomes and 0.943 (95% CI 0.943–0.944) for hospitalization, which were higher than those of the conventional triage system.

**Conclusions:**

The machine learning-based model using structured triage data from a nationwide database can effectively predict critical illness and hospitalizations among children visiting the ED.

## Introduction

In the emergency department (ED), triage is the first and most important step and classifies patients according to acuity and severity [1]. However, triage classifications tend to be similar but not always identical to ED or hospitalization outcomes [2,3] because triage systems are designed to provide timely and appropriate treatment in a resource-limited ED environment [4–6], not to predict the clinical outcome of the patient. However, early identification of patients at risk of deterioration is also a topic of interest for many individuals. Thus, studies have tried to predict critical or hospitalization outcomes from EDs [7–12].

The Pediatric Early Warning Score (PEWS) is an example of a scoring system used to detect children who are in need of intensive care unit (ICU) admission [13]. PEWS was originally developed and validated in the inpatient setting [14,15], but some validation in the ED setting was attempted [16,17]. Another attempt at the hospitalization prediction scoring system is the Pediatric Risk of Admission (PRISA) score [11,12]. The PRISA score was developed to predict hospitalization in the pediatric ED, but this scoring system is composed of 21 components gathered after initial evaluation, including therapies, which makes it difficult to apply at the initial presentation to the ED.

However, machine learning began to augment medical research, and various studies have attempted to introduce prediction models using machine learning. Machine learning models, such as random forest (RF), gradient boosting, and deep neural network methods, are able to handle large datasets effectively and have been shown to predict clinical outcomes more accurately than traditional methods for patients in the ICU and patients with sepsis [18–22]. Additionally, some studies have demonstrated that machine learning models can offer advantages in predicting critical condition and hospitalization outcomes [23–27], even in the pediatric population [28,29].

In this study, we used nationwide data from the National Emergency Department Information System (NEDIS) to develop a machine learning-based classification model to predict the clinical course of pediatric ED visitors. We also compared the performance of the derived machine learning model with that of the conventional pediatric triage system of South Korea (pediatric Korean Triage and Acuity Scale [pedKTAS]). In addition, we sought to define the importance of factors that predict critical cases and hospitalization among the selected predictor variables used in the analysis.

## Methods

### Study design and setting

This is a cross-sectional observational study investigating pediatric patients visiting the ED in South Korea using nationwide registry data. The Korean Triage and Acuity Scale (KTAS) is a 5-level triage system (from level 1 the most critical to level 5 the nonurgent) that was developed based on the Canadian Triage and Acuity Scale (CTAS). This scale has been used since its introduction in 2016 and has shown adequate reliability and validity [30]. The KTAS is divided into adult and pedKTAS based on an age cutoff of 15 years. We included emergency visits by children under 15 years of age from January 1, 2016, to December 31, 2017, which was after the pedKTAS was introduced and established in South Korea.

We obtained data from the NEDIS, which is a national database that was developed in 2004 and collects information from more than 400 EDs across South Korea. The NEDIS database contains various types of information, such as patient age, sex, type of insurance, means of transportation, level of consciousness at presentation, time variables (visit, discharge, and admission), and vital signs at presentation. The NEDIS also provides information about ED disposition and final outcomes of each ED visit (information regarding discharge, transfer, and death). All patients arriving at the ED must be enrolled in the system. All patient-related information from ED arrival to discharge from hospital is transferred automatically from each ED to a central server, and inaccurate data are filtered by a data processing system. NEDIS data are available upon formal request and provided by the National Emergency Medical Center (data acquisition number: N20192821211).

The Institutional Review Board of Seoul National University Hospital approved this study (IRB No. E-1909-098-1065) with a waiver of consent. Patients or the public were not involved in the design, conduct, reporting, or dissemination plans of our research.

### Outcomes

The primary outcome of this study was the prediction of critically ill children (critical cases) from triage data. Critical cases were defined as 1) children who were admitted to the ICU or transferred for ICU admission, 2) children who received cardiopulmonary resuscitation during their ED stay, and 3) children who died in the ED. The secondary outcome was the identification of children who could not be discharged directly from the ED (hospitalization) from triage data. Hospitalization was defined as including both admission to ICUs and general wards.

### Predictor variables and preprocessing

Demographic information, such as patient age and sex, was collected. Vital signs (blood pressure, heart rate, respiratory rate, body temperature and oxygen saturation) and consciousness level measured on the AVPU scale (alert, verbal response, response to pain, and unresponsive) at triage, transportation method, reason for ED visit (traumatic or nontraumatic), ED visit time and time from onset were also collected. A detailed list of variables used in the development of the model is shown in S1 Table.

Data on vital signs were preprocessed for machine learning because the normal values of some vital signs vary depending on age (such as blood pressure, heart rate, and respiratory rate). The Z scores of these age-dependent variables were calculated for each age range for adjustment before the final analysis. Categorical variables with low cardinality (sex and level of consciousness) were one-hot encoded. Missing values for continuous variables were imputed as the means of the nonmissing values of each corresponding variable, and missing values for categorical variables were coded as “Not Available”, representing an additional category (using one-hot encoding).

### Training of machine learning classifiers

In this study, we used RF to identify critically ill children and predict hospitalization. In machine learning (ML), algorithms are often not interpretable, the so-called “black box phenomenon”. However, compared to the “black box” models, interpretable models have shown technical equivalence [31,32]. The RF algorithm can calculate the importance of variables used in the model using reduction of the Gini index, thereby solving the “black box” problem and making the model more interpretable to some extent, allowing us to identify important variables. Therefore, we selected RF as the ML algorithm for the predictive model in this study.

Due to the imbalance in the entire dataset, the eligible study population was under sampled at a ratio of 1:1 in both critical cases and hospitalization cases using the python package ‘imbalanced-learn’ [33]. Each under sampled dataset was subjected to model derivation and testing through a 5-fold cross validation process. The “RandomForestClassifier” function of Python’s Scikit-Learn library was used for RF model development and testing. The default value of this function was used for the remaining the hyperparameters except for the number of trees (“n_estimator”). Regarding the number of trees, a value between 10 and 1000 showing excellent performance was used [34]. In addition, we also compared the performance of our models with that of pedKTAS, which served as the reference model.

### Data analysis

All data handling, statistical analysis and machine learning were performed with R platform version 3.6.3 (R Foundation for Statistical Computing, Vienna, Austria). Continuous variables were reported using medians and interquartile ranges (IQRs), and categorical variables were reported using frequencies and proportions.

The performance of the RF models was assessed by calculating the area under the receiver operating characteristic curve (AUROC) and the area under the precision-recall curve (AUPRC) with the 95% confidence interval (CI). In addition, the importance of each variable was calculated by decreasing the Gini index, and Scikit-Learn’s “feature importance” function was used [34].

## Results

A total of 2,621,710 ED visits were made by children younger than 15 years old and classified by the pedKTAS during the study period. As described above, the eligible cases were under sampled at a ratio of 1:1 to overcome imbalance for both the critical case group and hospitalization group. The basic demographic and clinical characteristics of the cases included in the analysis are summarized in Table 1. Overall, 12,951 (0.5%) patients had critical clinical outcomes (critical cases), and 303,808 (11.6%) patients were hospitalized (hospitalization). For the total population, the median age was 3.0 years old (IQR 1.0–7.0), and 57.2% of the children were male. Among the eligible patients, 22,359 (0.9%) had an unknown disease/injury category, and 71,976 (2.7%) had an unknown initial mental status.

**Table 1:** Demographic and clinical information of eligible cases.

Variable		Entire dataset (n = 2621710)	Under sampled dataset–Critical cases (n = 25902)	Under sampled dataset–Hospitalization (n = 607616)
Age, years		3.0 (1.0–7.0)	1.0 (0.0–5.0)	3.0 (1.0–6.0)
Sex	Female	1,122,069 (42.8)	10,988 (42.4)	259,029 (42.6)
	Male	1,499,641 (57.2)	14,914 (57.6)	348,587 (57.4)
Vital signs	Systolic blood pressure, mmHg	106.0 (99.0–118.0)	100.0 (90.0–117.0)	105.0 (97.0–118.0)
	Diastolic blood pressure, mmHg	64.0 (60.0–73.0)	60.0 (51.0–71.0)	63.0 (60.0–73.0)
	Heart rate, beats/minute	120.0 (100.0–133.0)	132.0 (110.0–154.0)	122.0 (102.0–140.0)
	Respiratory rate, breaths/minute	24.0 (20.0–28.0)	28.0 (22.0–36.0)	24.0 (20.0–29.0)
	Body temperature (°C)	37.0 (36.6–38.1)	37.0 (36.6–37.9)	37.2 (36.6–38.3)
	Percutaneous oxygen saturation (%)	99.0 (98.0–100.0)	99.0 (97.0–100.0)	99.0 (98.0–100.0)
Time from onset to ED visit, hours		4.0 (1.0–23.0)	4.0 (1.0–22.0)	7.0 (1.0–26.0)
Region	Metropolitan	612,491 (23.4)	5,836 (22.5)	141,560 (23.3)
	Urban	602,223 (23.0)	6,334 (24.5)	144,819 (23.8)
	Rural	1,406,996 (53.7)	13,732 (53.0)	321,237 (52.9)
EMC class	Regional EMC	879,240 (33.5)	10,767 (41.6)	219,550 (36.1)
	Local EMC	1,666,350 (63.6)	14,692 (56.7)	373,201 (61.4)
	Local ED	74,996 (2.9)	428 (1.7)	14,623 (2.4)
	Others	1,124 (0.0)	15 (0.1)	242 (0.0)
Disease or injury	Disease	1,851,355 (70.6)	20,148 (77.8)	480,586 (79.1)
	Injury	747,996 (28.5)	5,587 (21.6)	122,443 (20.2)
	Unknown	22,359 (0.9)	167 (0.6)	4,587 (0.8)
Mental status	Alert	2,534,339 (96.7)	23,054 (89.0)	583,934 (96.1)
	Verbal responsive	8,769 (0.3)	542 (2.1)	4,416 (0.7)
	Pain responsive	4,878 (0.2)	789 (3.0)	3,540 (0.6)
	Unresponsive	1,748 (0.1)	1,097 (4.2)	1,563 (0.3)
	Unknown	71,976 (2.7)	420 (1.6)	14,163 (2.3)
pedKTAS level	1	5641 (0.2)	1696 (6.5)	4561 (0.8)
	2	102,490 (3.9)	6,775 (26.2)	50,790 (8.4)
	3	823,154 (31.4)	7,101 (27.4)	233,813 (38.5)
	4	1,449,461 (55.3)	8,698 (33.6)	277,793 (45.7)
	5	240,964 (9.2)	1,632 (6.3)	40,659 (6.7)
ED disposition	Mortality	763 (0.0)	763 (2.9)	763 (0.1)
	Admission to ICU	12,188 (0.5)	12,188 (47.1)	12,188 (2.0)
	Admission to GW	290,857 (11.1)	1,404 (5.4)	290,857 (47.9)
	Discharge	2,317,902 (88.4)	11,547 (44.6)	303,808 (50.0)
Critical case	Yes	12,951 (0.5)	12,951 (50.0)	12,951 (2.1)
	No	2,608,759 (99.5)	12,951 (50.0)	594,665 (97.9)
Hospitalization	Yes	303,808 (11.6)	14,355 (55.4)	303,808 (50.0)
	No	2,317,902 (88.4)	11,547 (44.6)	303,808 (50.0)

EMC = Emergency medical center, ED = Emergency department, NA = Not Applicable, EM = Emergency medicine, pedKTAS = Pediatric Korean Triage and Acuity Scale, ICU = Intensive care unit, and GW = General ward.

**Fig 1:**
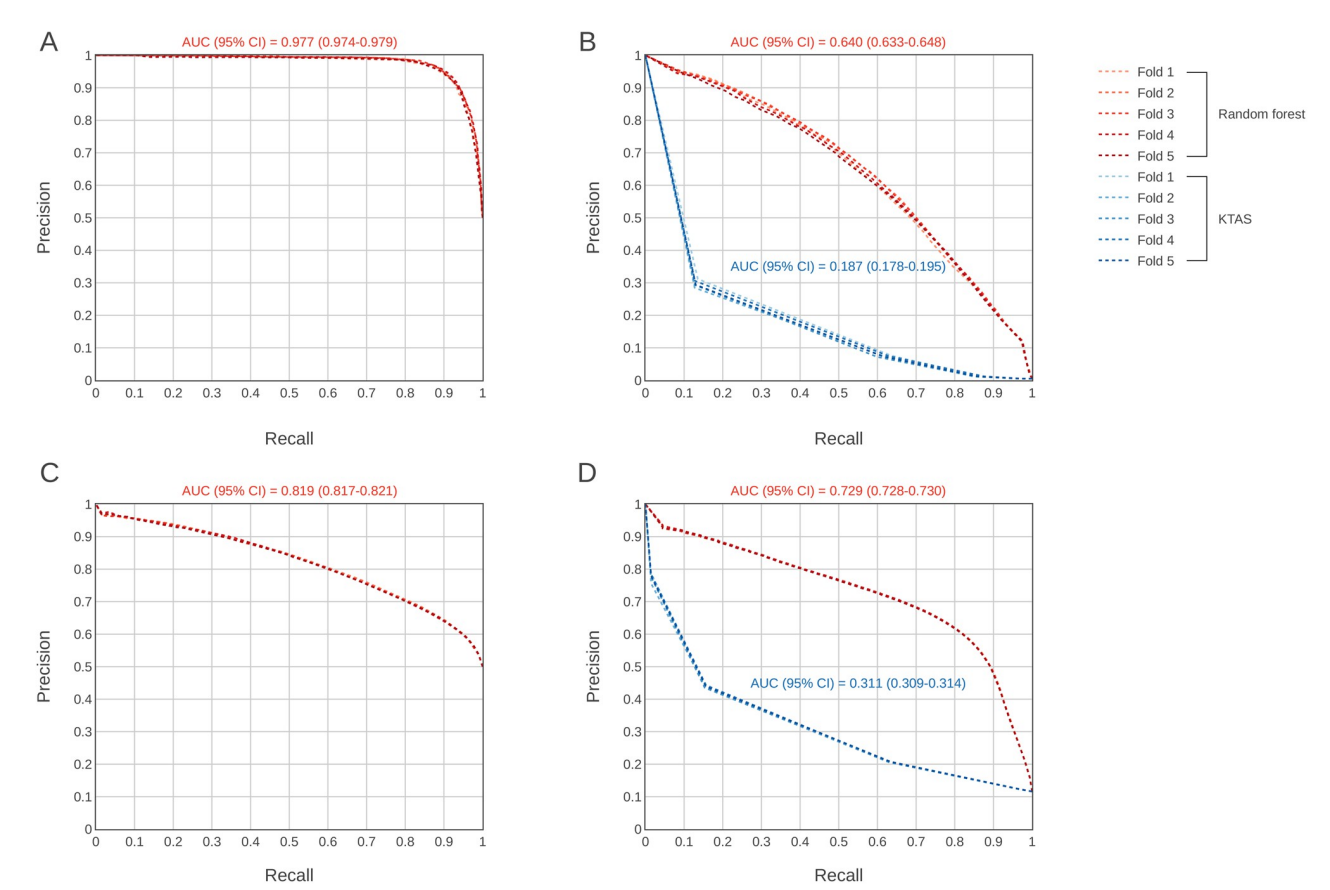
Receiver operating characteristic curves comparing the performance of the random forest model with that of the conventional triage system (pedKTAS). A. Under sampled dataset—critical cases, B. Validation with the entire dataset compared with pedKTAS–critical cases, C. Under sampled dataset–hospitalization, D. Validation with the entire dataset compared with pedKTAS–hospitalization. AUC = Area under the curve, CI = Confidence interval.

Fig 1 presents the classification results of the RF models and the comparison with pedKTAS for both outcomes. For the prediction of critical cases, the AUROC was 0.973 (95% CI 0.971–0.977) in the under sampled dataset (Fig 1A) and 0.991 (95% CI 0.991–0.992) in the validation dataset (Fig 1B). For the prediction of hospitalization, the AUROC was 0.819 in the under sampled dataset (Fig 1C) and 0.943 (95% CI 0.943–0.944) in the validation dataset (Fig 1D). For validation with the entire dataset, we also compared the prediction performance with that of pedKTAS.

**Fig 2:**
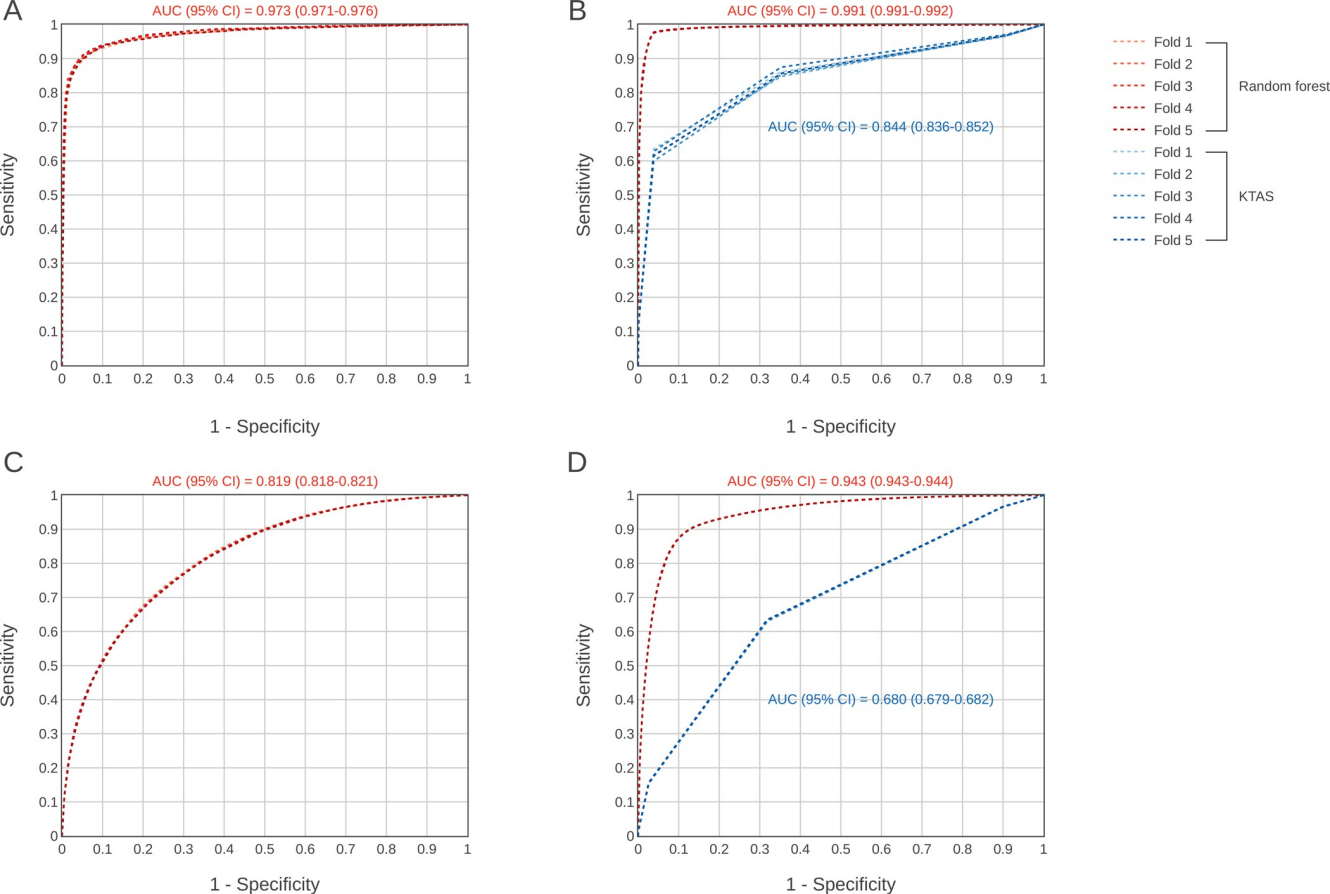
Precision-recall curves comparing the performance of the random forest model with that of the conventional triage system (pedKTAS). A. Under sampled dataset—critical cases, B. Validation with the entire dataset compared with pedKTAS–critical cases, C. Under sampled dataset–hospitalization, D. Validation with the entire dataset compared with pedKTAS–hospitalization. AUC = Area under the curve, CI = Confidence interval.

Additionally, the AUPRC of RF models for the under sampled dataset and validation with the entire dataset, including comparison with pedKTAS, are shown in Fig 2. The AUPRC was 0.977 (95% CI 0.974–0.979) in the under sampled dataset and 0.640 (95% CI 0.633–0.648) in the validation dataset (Fig 2B). However, the AUPRC was 0.819 (95% CI 0.817–0.821) in the under sampled dataset and 0.729 (95% CI 0.728–0.73) in the validation dataset. In validation, the performance was compared with that of the conventional triage system (pedKTAS).

**Fig 3:**
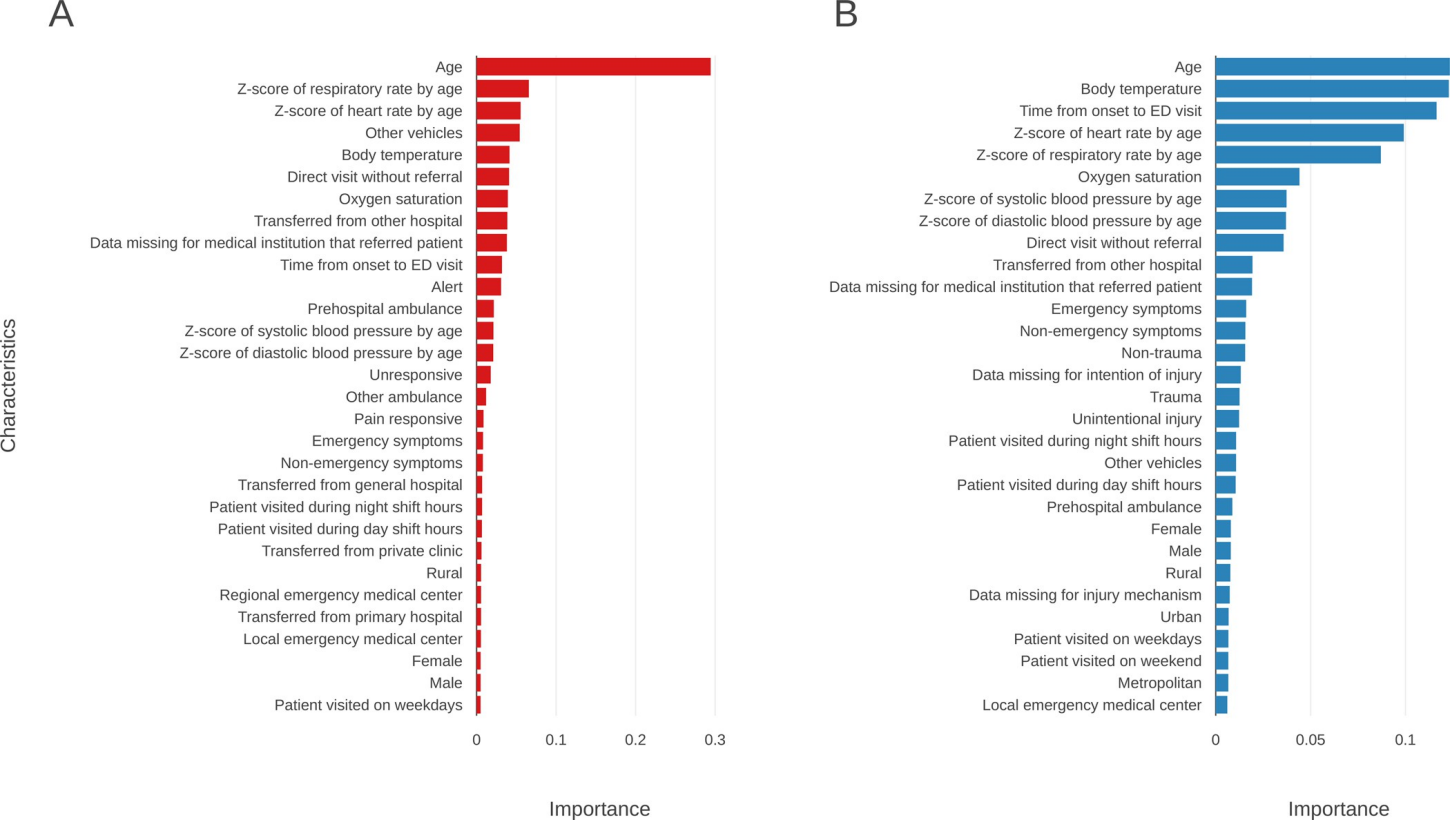
Top thirty predictors with the highest importance for each outcome. The importance of each feature was calculated through information gain using the difference in Gini impurity reduction. The “feature importance” function of Python’s scikit-learn library was used [34]. A. Critical cases, and B. hospitalization.

Fig 3 graphically displays the predictor variable importance. For critical cases, age was the most important variable followed by respiratory rate, heart rate, arrival at other vehicles, and body temperature. For hospitalization, age was also the most important variable with body temperature being a close second. The other important variables for hospitalization were time from onset to ED visit, heart rate, and respiratory rate.

## Discussion

In this study, using national data from 2,621,710 ED visits by children, we developed and compared several ML models to predict critical cases and hospitalization upon arrival at the ED. The RF model presented good performance discriminating critical cases with an AUROC of 0.991 and AUPRC of 0.640 from the limited information provided at the initial presentation. To our knowledge, this is the first attempt to use an ML algorithm for predicting outcomes in pediatric ED visitors using nationwide data. The RF model achieved higher performance than the conventional clinical prediction rules in predicting both critical cases and hospitalization among pediatric ED visitors with AUROC values of 0.991 versus 0.844 and 0.943 versus 0.680, respectively. These scoring systems have better performance than pre-existing scoring systems for clinical prediction [10,17,35]. These conventional methods consist of fewer variables and use a linear model with few interactions, whereas ML can perform high-order calculations.

In a previous study by Goto et al [28] that predicted pediatric outcomes in ED triage based on an ML model, the performance of the RF model had an AUROC of 0.85 (95% CI 0.79–0.91) for critical cases and 0.80 (95% CI 0.78–0.81) for hospital admissions. The improvement in the AUROC in our study may be due to the greater number of predictor variables used in our analysis. Additionally, there were slight differences in the choice of variables. In the abovementioned study, the important predictors for critical care included age, vital signs, and arrival mode. In contrast, our study showed a similar pattern for the importance of variables, except for ‘level of consciousness’ and ‘time from onset to ED visit’. These variables were of high importance in our study and were not included in the analysis of the previous study.

In another study, a gradient boosting model was used to predict mortality in an adult population with AUROC values ranging from 0.949–0.960 [36]. Give that this study was conducted in a single institute, it was possible to obtain more detailed variables, such as ‘unstructured chief complaint’ or ‘number of days to previous ED visit’. Our study used only highly quality-controlled and structured data, which did not completely utilize the various abilities of ML. Integration of unstructured data, such as text data, into the algorithm may present new possibilities. In addition to the abovementioned study, Choi et al. [37] showed that the addition of text data improves the predictive performance of ML triage compared to that of a model using only structured data. Lucini et al. [38] predicted the need for hospitalization based on written records of the first medical assessment in the ED using text-mining approaches.

There are some limitations of our study. First, although our model showed high AUROC values of 0.991 (for critical cases) and 0.943 (for hospitalization), the AUPRC of the entire dataset was low (0.640 for critical cases and 0.729 for hospitalization), which was probably due to the imbalanced dataset [39]. Critical cases accounted for only 0.5% (n = 12,951) of the total population, and we tried to overcome imbalance using the under sampling method. With under sampled training data, the AUPRC was higher (0.977 for critical cases and 0.819 for hospitalization) than the validation with the entire dataset. In predicting hospitalization, the RF model showed a lower AUROC than predicting critical cases. Moreover, it showed better AUPRC than AUPRC for predicting critical cases, which was probably due to a larger number of children being hospitalized (n = 303,808).

Second, although we used nationwide data, some bias is possible. As mentioned in the methods, some variables has missing values that we had to impute to classify as ‘unknown’. Additionally, input errors from each hospital could occur. However, the NEDIS dataset is quality-controlled by the National Emergency Medical Center of Korea and regularly undergoes a quality assessment process [40,41].

Finally, although this study used a large dataset from a nationwide registry, further studies in other countries and/or prospective validation must be performed. However, for the prospective validation of our ML model, the development of an EMR-embedded program with automatic calculation will be appropriate and must precede the experiment.

## Conclusions

ML models using structured triage data from a nationwide database can more effectively predict critical cases and hospitalizations among pediatric ED visitors than the conventional triage method. Age was the most important predictor for both ED outcomes, but importance of the other predictors differs between critical cases and hospitalization. Although prospective validation and integration of unstructured data are needed, the results of this study can support advances in pediatric triage and resource distribution in PED.

## Supporting information

S1 Table Variables used to develop the random forest model. (DOCX)

## References

[1] Williams RM. Triage and emergency department services. Ann Emerg Med. 1996;27(4):506–8. Epub 1996/04/01. doi: 10.1016/s0196-0644(96)99999-0 . 8604871

[2] Becker JB, Lopes MC, Pinto MF, Campanharo CR, Barbosa DA, Batista RE. [Triage at the Emergency Department: association between triage levels and patient outcome]. Rev Esc Enferm USP. 2015;49(5):783–9. Epub 2015/10/31. doi: 10.1590/S0080-623420150000500011 . 26516748

[3] Lim YD, Lee DH, Lee BK, Cho YS, Choi G. Validity of the Korean Triage and Acuity Scale for predicting 30-day mortality due to severe trauma: a retrospective single-center study. Eur J Trauma Emerg Surg. 2020;46(4):895–901. Epub 20181119. doi: 10.1007/s00068-018-1048-y . 30456416

[4] Murray M, Bullard M, Grafstein E, Group CNW, Group CNW. Revisions to the Canadian Emergency Department Triage and Acuity Scale implementation guidelines. CJEM. 2004;6(6):421–7. Epub 2007/03/24. . 17378961

[5] Green NA, Durani Y, Brecher D, DePiero A, Loiselle J, Attia M. Emergency Severity Index version 4: a valid and reliable tool in pediatric emergency department triage. Pediatr Emerg Care. 2012;28(8):753–7. Epub 2012/08/04. doi: 10.1097/PEC.0b013e3182621813 . 22858740

[6] Park J, Lim T. Korean Triage and Acuity Scale (KTAS). Journal of The Korean Society of Emergency Medicine. 2017;28(6):547–51.

[7] Shapiro NI, Wolfe RE, Moore RB, Smith E, Burdick E, Bates DW. Mortality in Emergency Department Sepsis (MEDS) score: a prospectively derived and validated clinical prediction rule. Crit Care Med. 2003;31(3):670–5. doi: 10.1097/01.CCM.0000054867.01688.D1 . 12626967

[8] Goto Y, Maeda T, Goto Y. Decision-tree model for predicting outcomes after out-of-hospital cardiac arrest in the emergency department. Crit Care. 2013;17(4):R133. Epub 20130711. doi: 10.1186/cc12812 ; PubMed Central PMCID: PMC4057027. 23844724 PMC4057027

[9] Barak-Corren Y, Fine AM, Reis BY. Early Prediction Model of Patient Hospitalization From the Pediatric Emergency Department. Pediatrics. 2017;139(5):e20162785. Epub 2017/05/31. doi: 10.1542/peds.2016-2785 . 28557729

[10] Egdell P, Finlay L, Pedley DK. The PAWS score: validation of an early warning scoring system for the initial assessment of children in the emergency department. Emerg Med J. 2008;25(11):745–9. Epub 2008/10/29. doi: 10.1136/emj.2007.054965 . 18955610

[11] Chamberlain JM, Patel KM, Ruttimann UE, Pollack MM. Pediatric risk of admission (PRISA): a measure of severity of illness for assessing the risk of hospitalization from the emergency department. Ann Emerg Med. 1998;32(2):161–9. doi: 10.1016/s0196-0644(98)70132-5 . 9701299

[12] Gravel J, Gouin S, Amre D, Bergeron S, Lacroix J. Evaluation of the pediatric risk of admission score in a pediatric emergency department. Ann Emerg Med. 2003;41(5):630–8. doi: 10.1067/mem.2003.139 . 12712029

[13] Seiger N, Maconochie I, Oostenbrink R, Moll HA. Validity of different pediatric early warning scores in the emergency department. Pediatrics. 2013;132(4):e841–50. Epub 20130909. doi: 10.1542/peds.2012-3594 . 24019413

[14] Duncan H, Hutchison J, Parshuram CS. The Pediatric Early Warning System score: a severity of illness score to predict urgent medical need in hospitalized children. J Crit Care. 2006;21(3):271–8. Epub 2006/09/23. doi: 10.1016/j.jcrc.2006.06.007 . 16990097

[15] Akre M, Finkelstein M, Erickson M, Liu M, Vanderbilt L, Billman G. Sensitivity of the pediatric early warning score to identify patient deterioration. Pediatrics. 2010;125(4):e763–9. Epub 20100322. doi: 10.1542/peds.2009-0338 . 20308222

[16] Breslin K, Marx J, Hoffman H, McBeth R, Pavuluri P. Pediatric early warning score at time of emergency department disposition is associated with level of care. Pediatr Emerg Care. 2014;30(2):97–103. Epub 2014/01/25. doi: 10.1097/PEC.0000000000000063 . 24457497

[17] Gold DL, Mihalov LK, Cohen DM. Evaluating the Pediatric Early Warning Score (PEWS) system for admitted patients in the pediatric emergency department. Acad Emerg Med. 2014;21(11):1249–56. Epub 2014/11/08. doi: 10.1111/acem.12514 ; PubMed Central PMCID: PMC4300231. 25377402 PMC4300231

[18] Kim J, Chang H, Kim D, Jang DH, Park I, Kim K. Machine learning for prediction of septic shock at initial triage in emergency department. J Crit Care. 2020;55:163–70. Epub 20191022. doi: 10.1016/j.jcrc.2019.09.024 . 31734491

[19] Vistisen ST, Johnson AEW, Scheeren TWL. Predicting vital sign deterioration with artificial intelligence or machine learning. J Clin Monit Comput. 2019;33(6):949–51. Epub 20190628. doi: 10.1007/s10877-019-00343-7 . 31254239

[20] Shillan D, Sterne JAC, Champneys A, Gibbison B. Use of machine learning to analyse routinely collected intensive care unit data: a systematic review. Crit Care. 2019;23(1):284. Epub 20190822. doi: 10.1186/s13054-019-2564-9 ; PubMed Central PMCID: PMC6704673. 31439010 PMC6704673

[21] Horng S, Sontag DA, Halpern Y, Jernite Y, Shapiro NI, Nathanson LA. Creating an automated trigger for sepsis clinical decision support at emergency department triage using machine learning. PLoS One. 2017;12(4):e0174708. Epub 20170406. doi: 10.1371/journal.pone.0174708 ; PubMed Central PMCID: PMC5383046. 28384212 PMC5383046

[22] Churpek MM, Yuen TC, Winslow C, Meltzer DO, Kattan MW, Edelson DP. Multicenter Comparison of Machine Learning Methods and Conventional Regression for Predicting Clinical Deterioration on the Wards. Crit Care Med. 2016;44(2):368–74. Epub 2016/01/16. doi: 10.1097/CCM.0000000000001571 ; PubMed Central PMCID: PMC4736499. 26771782 PMC4736499

[23] Levin S, Toerper M, Hamrock E, Hinson JS, Barnes S, Gardner H, et al. Machine-Learning-Based Electronic Triage More Accurately Differentiates Patients With Respect to Clinical Outcomes Compared With the Emergency Severity Index. Ann Emerg Med. 2018;71(5):565–74 e2. Epub 20170906. doi: 10.1016/j.annemergmed.2017.08.005 . 28888332

[24] Raita Y, Goto T, Faridi MK, Brown DFM, Camargo CA Jr., Hasegawa K. Emergency department triage prediction of clinical outcomes using machine learning models. Crit Care. 2019;23(1):64. Epub 20190222. doi: 10.1186/s13054-019-2351-7 ; PubMed Central PMCID: PMC6387562. 30795786 PMC6387562

[25] Hong WS, Haimovich AD, Taylor RA. Predicting hospital admission at emergency department triage using machine learning. PLoS One. 2018;13(7):e0201016. Epub 20180720. doi: 10.1371/journal.pone.0201016 ; PubMed Central PMCID: PMC6054406. 30028888 PMC6054406

[26] Ong ME, Lee Ng CH, Goh K, Liu N, Koh ZX, Shahidah N, et al. Prediction of cardiac arrest in critically ill patients presenting to the emergency department using a machine learning score incorporating heart rate variability compared with the modified early warning score. Crit Care. 2012;16(3):R108. Epub 20120621. doi: 10.1186/cc11396 ; PubMed Central PMCID: PMC3580666. 22715923 PMC3580666

[27] Kwon JM, Lee Y, Lee Y, Lee S, Park H, Park J. Validation of deep-learning-based triage and acuity score using a large national dataset. PLoS One. 2018;13(10):e0205836. Epub 20181015. doi: 10.1371/journal.pone.0205836 ; PubMed Central PMCID: PMC6188844. 30321231 PMC6188844

[28] Goto T, Camargo CA Jr., Faridi MK, Freishtat RJ, Hasegawa K. Machine Learning-Based Prediction of Clinical Outcomes for Children During Emergency Department Triage. JAMA Netw Open. 2019;2(1):e186937. Epub 20190104. doi: 10.1001/jamanetworkopen.2018.6937 ; PubMed Central PMCID: PMC6484561. 30646206 PMC6484561

[29] Patel SJ, Chamberlain DB, Chamberlain JM. A Machine Learning Approach to Predicting Need for Hospitalization for Pediatric Asthma Exacerbation at the Time of Emergency Department Triage. Acad Emerg Med. 2018;25(12):1463–70. Epub 20181129. doi: 10.1111/acem.13655 . 30382605

[30] Kim JH, Kim JW, Kim SY, Hong DY, Park SO, Baek KJ, et al. Validation of the Korean Triage and Acuity Scale Compare to Triage by Emergency Severity Index for Emergency Adult Patient: Preliminary Study in a Tertiary Hospital Emergency Medical Center. Journal of The Korean Society of Emergency Medicine. 2016;27(5):436–41.

[31] Rudin C. Stop explaining black box machine learning models for high stakes decisions and use interpretable models instead. Nature Machine Intelligence. 2019;1(5):206–15. doi: 10.1038/s42256-019-0048-x WOS:000567063700004. PMC9122117 35603010

[32] Rudin C, Radin J. Why Are We Using Black Box Models in AI When We Don’t Need To? A Lesson From An Explainable AI Competition. Harvard Data Science Review. 2019;1(2). doi: 10.1162/99608f92.5a8a3a3d

[33] Lemaître G, Nogueira F, Aridas CK. Imbalanced-learn: a python toolbox to tackle the curse of imbalanced datasets in machine learning. J Mach Learn Res. 2017;18(1):559–63.

[34] Pedregosa F, Varoquaux G, Gramfort A, Michel V, Thirion B, Grisel O, et al. Scikit-learn: Machine Learning in Python. Journal of Machine Learning Research. 2011;12:2825–30. WOS:000298103200003.

[35] Chamberlain JM, Patel KM, Pollack MM, Brayer A, Macias CG, Okada P, et al. Recalibration of the pediatric risk of admission score using a multi-institutional sample. Ann Emerg Med. 2004;43(4):461–8. doi: 10.1016/j.annemergmed.2003.08.001 . 15039688

[36] Klug M, Barash Y, Bechler S, Resheff YS, Tron T, Ironi A, et al. A Gradient Boosting Machine Learning Model for Predicting Early Mortality in the Emergency Department Triage: Devising a Nine-Point Triage Score. J Gen Intern Med. 2020;35(1):220–7. Epub 20191101. doi: 10.1007/s11606-019-05512-7 ; PubMed Central PMCID: PMC6957629. 31677104 PMC6957629

[37] Choi SW, Ko T, Hong KJ, Kim KH. Machine Learning-Based Prediction of Korean Triage and Acuity Scale Level in Emergency Department Patients. Healthc Inform Res. 2019;25(4):305–12. Epub 20191031. doi: 10.4258/hir.2019.25.4.305 ; PubMed Central PMCID: PMC6859273. 31777674 PMC6859273

[38] Lucini FR, Fogliatto FS, da Silveira GJC, Neyeloff JL, Anzanello MJ, Kuchenbecker RS, et al. Text mining approach to predict hospital admissions using early medical records from the emergency department. Int J Med Inform. 2017;100:1–8. Epub 20170105. doi: 10.1016/j.ijmedinf.2017.01.001 . 28241931

[39] Saito T, Rehmsmeier M. The precision-recall plot is more informative than the ROC plot when evaluating binary classifiers on imbalanced datasets. PloS one. 2015;10(3):e0118432–e. doi: 10.1371/journal.pone.0118432 . 25738806 PMC4349800

[40] Kim TH, Hong KJ, Shin SD, Park GJ, Kim S, Hong N. Forecasting respiratory infectious outbreaks using ED-based syndromic surveillance for febrile ED visits in a Metropolitan City. Am J Emerg Med. 2019;37(2):183–8. Epub 20180510. doi: 10.1016/j.ajem.2018.05.007 ; PubMed Central PMCID: PMC7126969. 29779674 PMC7126969

[41] Kim JS, Seo DW, Kim YJ, Jeong J, Kang H, Han KS, et al. Prolonged Length of Stay in the Emergency Department and Increased Risk of In-Hospital Cardiac Arrest: A nationwide Population-Based Study in South Korea, 2016–2017. J Clin Med. 2020;9(7):2284. Epub 20200718. doi: 10.3390/jcm9072284 ; PubMed Central PMCID: PMC7408893. 32708363 PMC7408893

