# Supplementary material for: Machine learning-based prediction of critical illness in children visiting the emergency department
Source: PLoS One. 2022 Feb 17;17(2):e0264184. doi: 10.1371/journal.pone.0264184 (PMC8853514; doi:10.1371/journal.pone.0264184)
Supplement: S1 Table — (DOCX) [file pone.0264184.s001.docx]

Table S1. Variables used in the development of random forest model.

| Age, years | |
| --- | --- |
| Sex | Female |
|  | Male |
| Vital signs | Systolic blood pressure, mmHg |
|  | Diastolic blood pressure, mmHg |
|  | Heart rate, beats/minute |
|  | Respiratory rate, breaths/minute |
|  | Body temperature (℃) |
|  | Percutaneous oxygen saturation (%) |
| Time from onset to ED visit, hours | |
| Region | Metropolitan |
|  | Urban |
|  | Rural |
| EMC class | Regional EMC |
|  | Local EMC |
|  | Local ED |
|  | Others |
| Disease or injury | Disease |
|  | Injury |
|  | Unknown |
| Mental status | Alert |
|  | Verbal responsive |
|  | Pain responsive |
|  | Unresponsive |
|  | Unknown |
| KTAS level | 1 |
|  | 2 |
|  | 3 |
|  | 4 |
|  | 5 |
| ED disposition | Mortality |
|  | Admission to ICU |
|  | Admission to GW |
|  | Discharge |
| Critical case | Yes |
|  | No |
| Hospitalization | Yes |
|  | No |
| Intentionality of injury | Accidental, unintentional |
|  | Intentional self-harm, suicide |
|  | Violence, assault |
|  | Unspecified |
| Injury mechanism | Car accident |
|  | Bicycle accident |
|  | Motorcycle accident |
|  | Other traffic accident |
|  | Unspecified traffic accident |
|  | Fall from height |
|  | Slip down |
|  | Struck by blunt object |
|  | Penetrating injury |
|  | Injury due to industrial machinery |
|  | Burn |
|  | Drowning |
|  | Poisoning |
|  | Choking/hanging |
|  | Data missing for injury mechanism |
| Route of entry | Direct visit without referral |
|  | Transferred from other hospital |
|  | Referred from outpatient department |
|  | Visit with other specified referral information |
|  | Data missing for referral information |
| Transportation | Prehospital ambulance |
|  | Interhospital ambulance |
|  | Other ambulance |
|  | Public vehicles |
|  | Air transport |
|  | Other vehicles |
|  | Walk in |
|  | No information on transportation |
| Emergency | Emergency symptoms |
|  | Non-emergency symptoms |
|  | Data missing for emergency symptoms |
| Occupation of triage provider | Triage is performed by emergency medicine specialist |
|  | Triage is performed by emergency medicine resident |
|  | Triage is performed by intern |
|  | Triage is performed by general physician |
|  | Triage is performed by nurse |
|  | Triage is performed by paramedic |
|  | Data missing for triage provider |
| Day shift or night shift | Patient visited during day shift hours |
|  | Patient visited during night shift hour |
| Weekday | Patient visited on weekdays |
|  | Patient visited on weekend |
| Insurance | National health insurance |
|  | Automobile insurance |
|  | Industrial accident insurance |
|  | Medical aid type I |
|  | Medical aid type II |
|  | Uninsured |
|  | Other insurance |
|  | Data missing for insurance |
| Role of victim | Pedestrian |
|  | Driver |
|  | Passenger |
|  | Stewardess |
|  | Role information of the victim is unknown |
|  | Data missing for the role of the victim |
| Information of the medical institution that has been sent | Transferred from tertiary general hospital |
|  | Transferred from general hospital |
|  | Transferred from primary hospital |
|  | Transferred from private clinic |
|  | Transferred from oriental medical clinic |
|  | Transferred from medical institution with unknown name |
|  | Transferred from other medical institution |
|  | Data missing for medical institution that referred patient |

EMC = Emergency medical center, ED = Emergency department, NA = Not Applicable, EM = Emergency medicine, KTAS = Korean Triage and Acuity Scale, ICU = Intensive care unit, and GW = General ward
